# Planning and governing nature-based solutions in river landscapes: Concepts, cases, and insights

**DOI:** 10.1007/s13280-021-01569-z

**Published:** 2021-06-08

**Authors:** Christian Albert, Jochen Hack, Stefan Schmidt, Barbara Schröter

**Affiliations:** 1 Institute of Geography, Ruhr University Bochum, Universitaetsstr. 150, 44801 Bochum, Germany; 2 Section of Ecological Engineering, Institute of Applied Geosciences, Technical University Darmstadt, Schnittspahnstr. 9, 64287 Darmstadt, Germany; 3 Leibniz Centre for Agricultural Landscape Research (ZALF), Working Group “Governance of Ecosystem Services”, Eberswalder Str. 84, 15374 Müncheberg, Germany; 4 Centre for Sustainability Studies, Lund University, Box 170, 221 00 Lund, Sweden

**Keywords:** Effects, Governance, Landscape planning, Green infrastructure, Social–ecological research, Spatial planning

## Abstract

Nature-based solutions (NBS), understood as actions that use ecosystem processes to address societal needs, can play important roles to future-proof river landscape development for people and nature. However, knowledge gaps exist how NBS can be planned and implemented at landscape scales. This Special Issue brings together insights and experiences from studies of assessing, planning, and implementing NBS in river landscapes in Europe and beyond. It addresses three research fields: (i) NBS effects, looking at the effectiveness of NBS to achieve ecological, social, and/or economic outcomes, (ii) NBS planning, focusing on approaches for planning and designing NBS, and (iii) NBS governance, relating to governance and business models for implementation. The twelve contributions deliver evidence on how NBS outperform conventional, rather technical solutions, provide guidance and tools to operationalize the NBS concept into practice, and showcase successful governance models of NBS in different contexts. The editorial ends with an outlook on further research needs.

## Introduction

Since thousands of years, river landscapes across the world have been transformed by humans to increase their function as drainage, to enhance navigation, to produce energy, and to enable agricultural production and settlement in former floodplain areas (Brown et al. 2009, 2018; Posthumus et al. 2010). The degree of transformations exacerbated in the last two centuries has led to undesirable ecological effects over time, such as increased erosion, decrease, and pollution of groundwater resources, increase in flood probability, decline in fisheries and biodiversity, as well as loss of esthetics and recreational functions (Bunn and Arthington 2002; Malmqvist and Rundle 2002; Tockner et al. 2011; Sabater et al. 2018). To date, around 90% of Europe’s river landscapes have been changed (Tockner et al. 2011) and only 40% are in a good ecological condition (EEA 2018). In addition, changes in the river and flood regime as a result of climate change lead to further challenges (IPCC 2014; Pletterbauer et al. 2018), which entail considerable economic costs (Schäfer and Kowatsch 2015) and make it more difficult to sustain people’s quality of life (Vörösmarty et al. 2010; Kibria 2016).

In response to this unsustainable development, policy and decision makers have drawn up ambitious restoration programs to stop ecological degradation and to advance river landscape restoration. The Sustainable Development Goals 14 and 15 (Life on Land and Life below Water) explicitly aim at enhancing global ecological conditions of river landscapes. In the year 2000, the European Union issued the Water Framework Directive as a milestone policy program, aiming to achieve a ‘good status’ for all ground and surface waters including rivers in the EU by 2015, a target date that was later postponed to 2030. River restoration remains high on the political agenda, with the EU Biodiversity Strategy for 2030 recently calling to restore at least 25 000 km of rivers in the EU to a free-flowing state.

Nature-based solutions (NBS), i.e., activities inspired and supported by ecosystem processes to fulfill human and societal needs (European Commission 2015), can arguably play an important role in the emerging efforts to future-proof river landscape development for people and nature (Albert et al. 2019). NBS are seen as beneficial over purely technical solutions in that they minimize negative-side effects and instead create co-benefits for people and nature today and in the future (IUCN 2012; European Commission 2015; Davis et al. 2018). NBS today are considered important elements of international efforts to combat climate change (Nature-based Solutions Coalition 2019), to safeguard biodiversity (leaders pledge for nature, see leaderspledgefornature.org and Vaughan 2020), to advance ecosystem restoration (see decadeonrestoration.org), and, more recently, supportive approaches for advancing towards a green recovery in the aftermath of the SARS-COV-2 pandemic (e.g., Science for Environment Policy 2021).

Supported, among others, by targeted funding from the EU, research on NBS has grown dramatically, with the numbers of papers published on the subject rising from only three in 2015 to more than 250 in 2020 (April 7, 2021, Web of Science). While scientific debate about the exact definition and conceptualization of NBS continues (e.g., Eggermont et al. 2015; Albert et al. 2017; IUCN 2020), important advances have already been made and lessons learned of how NBS can be successfully planned and implemented (Short et al. 2018; Frantzeskaki 2019). In the context of river landscape development, recent research on NBS has addressed a wide range of challenges, including, for example, approaches to stormwater management (e.g., Pelorosso et al. 2018; Kopp et al. 2019; Simperler et al. 2020), flood risk management (Majidi et al. 2019; Pagano et al. 2019; Singh et al. 2020), and climate change mitigation and adaptation (e.g., Chausson et al. 2020; Wamsler et al. 2020). First special issues on NBS are also beginning to emerge, with early examples focusing on NBS for creating resourceful circular cities (Langergraber and Atanasova 2020), on NBS in cities in relation to justice and equity considerations (Sekulova et al. 2021) and on NBS for hydro-meteorological risk reduction (Lupp and Zingraff-Hamed 2021).

However, substantial knowledge gaps still exist, particularly on planning and implementation practices, effectiveness, and monitoring, as well as on governance aspects (Albert et al. 2019). This is particularly true for the application of NBS in the case of river landscapes which, compared to NBS in cities, has so far received comparatively less scientific attention. Practice examples are needed that showcase under which conditions NBS contribute to overcoming ecological, social, and economic challenges and how such solutions can be successfully planned and realized in different contexts (Cohen-Shacham et al. 2016). Especially in the Global South, more scientific evidence of NBS effects in different contexts is still needed (Chausson et al. 2020). Also, an enhanced understanding of NBS co-benefits is required for better exploiting synergetic solutions (Meerow et al. 2019). Further insights are required on how collaborations between different disciplines, stakeholders, and decision makers can be established and fostered to develop successful governance and business models for the implementation of NBS (Sekulova and Anguelovski 2017). Moreover, examples are needed that show how to integrate scientific, indigenous, and local knowledge to attune NBS to local contexts and enhance the likelihood of successful implementation (Hemmerling et al. 2020).

This Special Issue aims to bring together insights and experiences from studies of assessing, planning, and implementing NBS in river landscapes in Europe and beyond. More specifically, the Special Issue sheds light on scientific frontiers of NBS planning and governance in three relevant fields of research: (i) NBS effects, looking at the effectiveness of NBS to achieve ecological, social, and/or economic outcomes in river landscapes. (ii) NBS planning, focusing on methods, and insights concerning approaches for planning and designing NBS in river landscapes, and (iii) NBS governance, focusing on governance models for implementing preferred NBS, also including suitable business models. Although the Special Issue places a thematic focus on efforts for planning NBS in riverine ecosystems and at the landscape scale, the insights may also be instructive for NBS planning and governance efforts in other ecosystem types and at lower or higher levels of public and private decision making.

## Structure of the special issue

**Fig. 1:**
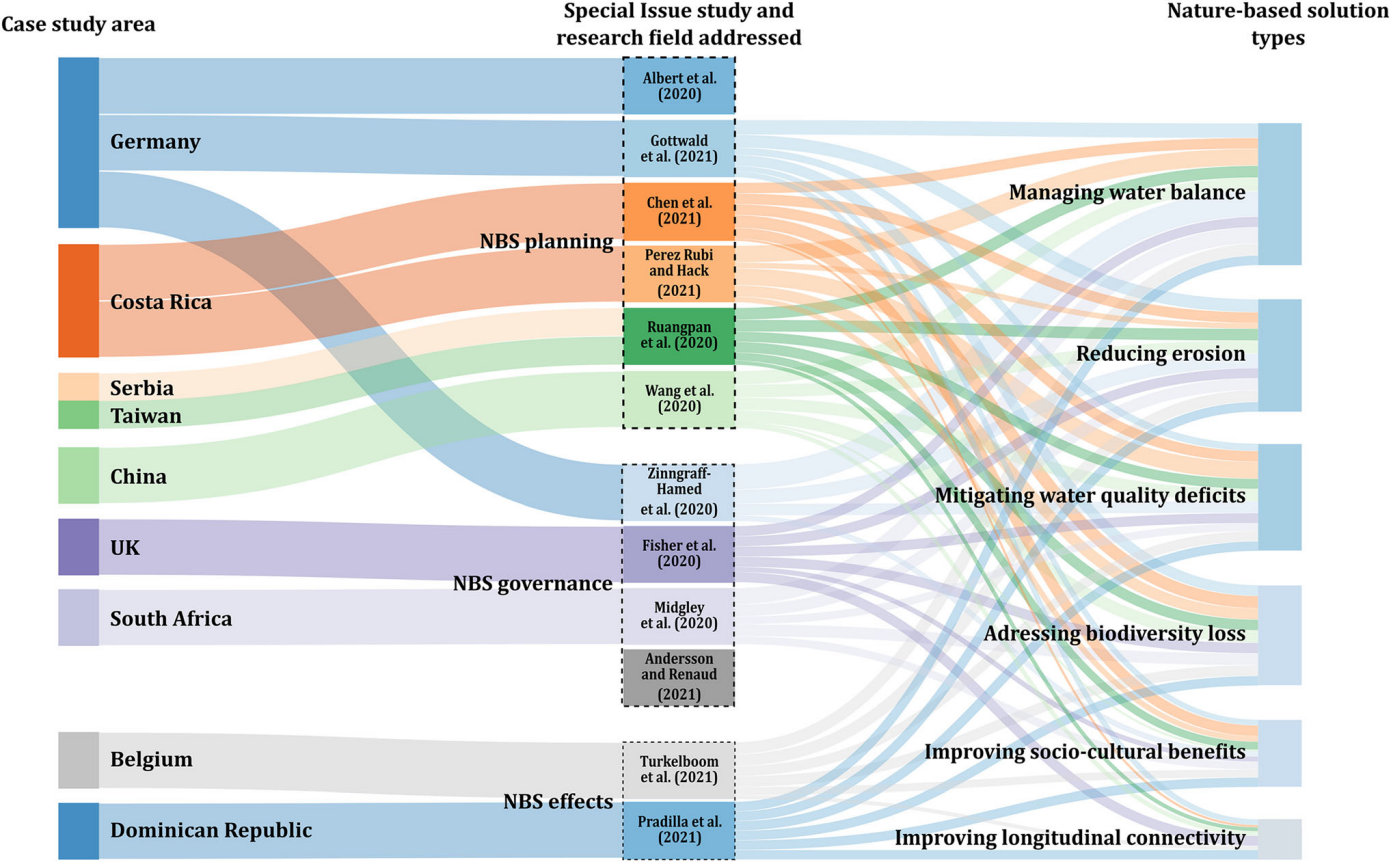
Contributions included in this Special Issue, their allocation to the three research fields, as well as case study areas and NBS types considered

The Special Issue contains a total of twelve manuscripts pertaining to three key fields of research. The first research field, NBS effects, is addressed by contributions by Pradilla et al. (2021) and Turkelbloom et al. (2021). NBS planning, the second research field, is covered by six manuscripts from Albert et al. (2021), Chen et al. (2021), Gottwald et al. (2021), Pérez-Rubi and Hack (2021), Ruangpan et al. (2021), and Wang et al. (2021). Finally, the third research on NBS governance features four contributions by Anderson and Renaud (2021), Fisher et al. (2021), Midgley et al. (2021), and Zingraff-Hamed et al. (2021). European case studies dominate and considered NBS relate mostly to measures that help to manage the water balance to address floods or droughts (Fig. 1, Table 1).

**Table 1:** Contributions included in the special issue

Topic	Authors	Paper title
NBS effects	Pradilla et al. (2021)	Hydromorphological and socio-cultural assessment of urban rivers to promote nature-based solutions in the Dominican Republic
	Turkelbloom et al. (2021)	How does a nature-based solution for flood control compare to a technical solution? Case study evidence from Belgium
NBS planning	Albert et al. (2021)	Planning nature-based solutions: Principles, steps, and insights
	Chen et al. (2021)	Development and Modelling of realistic retrofitted Nature-based Solution Scenarios to reduce Flood Occurrence at the Catchment Scale
	Gottwald et al. (2021)	Using Geodesign as a boundary management process for planning nature-based solutions in river landscapes
	Pérez-Rubi and Hack (2021)	Co-design of experimental Nature-based Solutions for decentralized dry-weather runoff treatment retrofitted in a densely urbanized area in Central America
	Ruangpan et al. (2021)	Incorporating stakeholders’ preferences into a multi-criteria framework for planning large-scale Nature-Based Solutions
	Wang et al. (2021)	Bridging the science-practice gaps in nature-based solutions: A riverfront planning in China
NBS governance	Anderson and Renaud (2021)	A review of public acceptance of Nature-based Solutions: the ‘why’, ‘when’, and ‘how’ of success for disaster risk reduction measures
	Fisher et al. (2021)	“It’s on the ‘nice to have´ pile”: Potential principles to improve the implementation of socially inclusive Green Infrastructure
	Midgley et al. (2021)	Typologies of collaborative governance for scalable nature-based solutions in two strategic South African river systems
	Zingraff-Hamed et al. (2021)	Governance models for nature-based solutions: Seventeen cases from Germany

### Part I: Effects of nature-based solutions in river landscapes

This first section of the Special Issue presents insights into the effects that NBS may yield in river landscapes. Turkelbloom et al. (2021) apply a case study research approach and social cost-benefit analysis in the Dijle river valley in central Belgium to assess and compare the effects of a nature-based and a technical alternative for flood damage protection. For the NBS option, they assumed a restoration of the alluvial floodplain, whereas the technical alternative was represented by an installation of a storm water basin.

Pradilla et al. (2021) explore the applicability of a socio-ecological assessment method to guide urban river restoration and strategic planning of NBS in Jarabacoa, Dominican Republic. The authors complemented a hydro-morphological assessment method widely used in Germany (LAWA-OS) with a citizen survey on the perception of blue-green infrastructure features of three streams in and near the town of Jarabacoa.

### Part II: Planning and designing nature-based solutions in river landscapes

The second part of the Special Issue brings together papers that present novel approaches for planning and designing NBS at the landscape scale. Albert et al. (2021) propose a conceptual framework for planning NBS at the landscape scale, consisting of three key criteria of NBS, six essential planning steps, and five overarching principles. The authors develop the framework based on a synthesis of relevant literature, report on an application in a transdisciplinary research project in the Lahn river landscape, Germany, and evaluate the degree to which the principles can be adhered to in real-world planning contexts.

Chen et al. (2021) explore decentralized NBS such as Urban Green Infrastructures (UGI) to reduce flooding in urban areas. Contrary to earlier studies which have shown the effectiveness of flood control of UGI at a plot or neighborhood level, this study, conducted in the metropolitan area of Costa Rica, proposes a scenario development and hydrological modeling approach for a more realistic upscaling of UGI by taking into account empirical insights from a representative neighborhood regarding the actual suitable space for UGI and potential implementation constraints.

Gottwald et al. (2021) develop and apply a novel Geodesign procedure in the planning with NBS in the Lahn river landscape in Hesse, Germany. A specific focus is put on the development of Geodesign tools that allow diverse stakeholders to interact with a spatial decision-support system in three planning phases: sketching and writing ideas, assigning land-use changes, and evaluating likely impacts. The Geodesign tools were applied in a workshop with stakeholders, facilitated by the use of a large touch table as an interface between participants and the digital, special decision-support system. Four ecosystem services were considered as criteria for evaluating impacts: food provision, climate change regulation, pollination, and recreation. In addition, a systematic evaluation was conducted to assess the contributions of the Geodesign exercise to the boundary management between participants.

Pérez-Rubi and Hack (2021) present an adaptive methodology for the design of NBS for decentralized urban runoff treatment in a Latin American context. Through this study, technical solutions commonly used for stormwater management were adapted for dry-weather runoff treatment and co-designed for the particular conditions of a representative study area, considering space availability as the main constraining factor for retrofitting in urban areas. By applying a co-design process in a densely urbanized neighborhood of the Great Metropolitan area of Costa Rica insights about conditions that could be hindering the implementation of NBS infrastructures in Latin America are intended to be revealed.

Ruangpan et al. (2021) are interested in the feasibility of measures for hydro-meteorological risk reduction. With a focus on potential NBS development options for reducing flood risks in the Tamnava river basis in Serbia and the Nangang river landscape in Taiwan, the authors develop and apply a multi-criteria assessment framework for a range of potentially feasible decision-making options.

Wang et al. (2021) strive to address the gap between science and practice in the field of NBS and propose planning as a bridging procedure. Focusing on a case study of the Jialing River in the Sichaun Province, China, the authors explore options for addressing three challenges: transforming riverfront planning towards holistic perspectives, effectively communicating the implications of NBS, and procedures for incorporating both scientific insights and local wisdom in plan and decision making.

### Part III: Realizing nature-based solutions with suitable governance

The third part for the Special Issue contains five contributions that deal with governance aspects of the implementation of NBS. The articles analyze social principles that decision makers should take into account, the inclusion of actor preferences and the stakeholder constellation per se in different governance models. These insights are important to overcome implementation barriers for NBS by improving institutions and carefully including all relevant actors important for NBS.

Anderson and Renaud (2021) perform a systematic review of the public acceptance of NBS. The authors compare technical and nature-based approaches to disaster risk reduction and try to identify factors of relevance for the acceptance of such approaches as relating to individuals and society in general.

Fisher et al. (2021) use a mixed method approach—a literature review and a survey among practitioners—to find out if social aspects really matter for the uptake of green infrastructure (GI) and are more than just something “nice to have.” The authors identify social principles guiding the implementation of GI in the United Kingdom and reflect on in how far these principles are taken into consideration in practice.

Midgley et al. (2021) aimed to explore how different collaborative governance models and financial arrangements play out in implemented NBS and how they can be upscaled to achieve greater impact. The authors developed an inventory that compiles actor, environmental, social, and financial dimensions and benefits of water-related ecological infrastructure intervention projects in two river systems in South Africa. By qualitatively and quantitatively analyzing the inventory, major characteristics of governance, financing and scalability could be revealed and scalable typologies identified that offer structures suited to increased investment.

Zingraff-Hamed et al. (2021) give an overview over implemented NBS for flood risk management and mitigation in Germany, and they combine a hierarchical clustering procedure and a qualitative analysis to identify governance models applied in 17 case studies and explore the differences between these models.

## Insights

### Nature-based solutions' effects in river landscapes

In comparison to conventional, technical solutions, NBS can perform equally while providing additional social and ecological benefits. A comprehensive assessment of potential effects of NBS and a comparison with alternative solutions should, therefore, be encouraged. Furthermore, taking into account stakeholder preferences and the variety of co-benefits can contribute to better decision making and NBS planning (Pradilla et al. 2021). However, certain preconditions, for instance the availability of space and its use as well as water quality, may be required to successfully implement and achieve benefits of NBS.

In the context of reducing flood risk through flood retention, it could be shown that NBS offer similar flood security, lower costs, more ecosystem services benefits, and higher biodiversity values than a technical option (Turkelbloom et al. 2021). When looking at urban flooding, NBS such as Urban Green Infrastructures can yield significant runoff reduction compared to conventional stormwater drainage when available space is effectively used (Chen et al. 2021). Chances for successful NBS implementation increase in conditions of sufficient space to retain flood water, when flood water is of sufficient quality, and when economic activity and housing in the floodplain are limited (Turkelbloom et al. 2021). The effects of NBS can be further enhanced when knowledge on co-benefits and stakeholders’ preferences enables decision makers in a multi-criteria assessment to identify the most suitable and preferable NBS measures for an area (Ruangpan et al. 2021). This contributes to the development of easy-to-use decision-support tools for planners and decision makers to enable a systematic and transparent NBS planning process.

### Nature-based solutions planning in river landscapes

Taken together, contributions to the section on planning NBS in river landscapes re-emphasize the important role that planning can take in operationalizing the NBS concept in practice and in facilitating processes of transdisciplinary knowledge co-generation.

First, the contributions show how planning with NBS in river landscapes could work across different scales. Albert et al. (2021) propose a framework of six essential steps for planning with NBS at landscape levels: Co-define setting, Understand challenges, Create visions and scenarios, Assess potential impacts, Develop solution strategies, and Realize and monitor. In addition, the authors propose five key principles to which the implementation of the planning steps should adhere to. Those principles are Place specificity, Evidence base, Integration, Equity, and Transdisciplinary. Drawing on insights from an empirical testing and evaluation of the planning steps in the Lahn case study, the authors find that adhering to those principles is possible through specific measures taken during the application of the planning steps, but that the degree to which the principles are implemented may vary over the course of a planning process. Chen et al. (2021) highlight the importance of taking space availability and site-specific constraints into account in order to generate plausible and relevant scenarios and impact assessments. In addition, they find that insights from detailed field work-based site assessments of a representative urban area can eventually be extrapolated to a larger watershed scale using a highly resolved land-use classification.

Second, contributions show how planning can facilitate the creation of novel plans with NBS to address societal challenges. For example, the application of a co-design process in a dense neighborhood of the Great Metropolitan area of Costa Rica (Pérez-Rubi and Hack 2021) enabled the development of strategic siting of NBS to address societal challenges of water treatment in dry-weather conditions. The chosen approach not only proved successful in devising suitable NBS but also brought to light the needs to carefully take into account implementation conditions and stakeholders demands early onwards during the planning process already.

And third, the contributions shed light on planning approaches that integrate diverse stakeholders in the planning process. Ruangpan et al. (2021) highlight the need to involve stakeholders in the early planning stages to achieve successful implementation of NBS. Local actors can introduce relevant data and considerations into the process of measure selection that might otherwise be disregarded by planners. One tool particularly supportive for such solution-oriented knowledge co-production can be Geodesign. In their pilot application in a workshop with local planners in the Lahn river landscape, Gottwald et al. (2021) found that Geodesign facilitated the co-design and exploration of NBS and contributed to boundary management between actors from different backgrounds. However, applying Geodesign in just one workshop alone is not sufficient but needs to be integrated within a larger collaborative planning and implementation processes. In a similar way, Wang et al. (2021) present how planning with NBS can help bridging top down and bottom-up planning and mediating between different stakeholders, thus, enhancing communication and expanding the planning goals towards multi-functionality.

### Nature-based solutions governance in river landscapes

The contributions to the section on governance of NBS in river landscapes stress that context and collaboration are key when improving governance aspects for NBS.

First, the spatial as well as the socio-ecological context matters. Turkelbloom et al. (2021) found that both are important for setting up a business case for NBS. Also, Pérez-Rubi and Hack (2021) highlighted the need for knowing and including context-specific conditions like stakeholder demands in the implementation of NBS. In a similar vein, Fisher et al. (2021) found that practitioners in the UK are in favor of incorporating social principles into the concept of GI that reach beyond the provision of socio-economic benefits: principles that ensured the inclusivity, equal access to, and long-term funding options for GI.

Second, the observation, establishment, and monitoring of public acceptance is neglected in governance processes but crucial to legitimize and sustain NBS infrastructures (Pérez-Rubi and Hack 2021). Especially long-term success of NBS consistently relies on a broader range of public acceptance outcomes (Anderson and Renaud 2021). Therefore, Anderson and Renaud (2021) propose a framework for understanding and increasing public acceptance of NBS. The model highlights the role of risk perception, trust, competing societal interests, and ecosystem services. Efforts to increase acceptance should focus on providing and promoting awareness of benefits combined with effective communication and collaboration (Anderson and Renaud 2021).

Third, collaboration is needed for implementing NBS. This is a logical consequence of the context importance but also a condition for upscaling NBS. Knowledge co-production and joint sense making with relevant landscape actors are essential for scaling NBS to larger areas and different contexts (Midgley et al. 2021). To take the context into account, different knowledge has to be considered, and different actors have to collaborate. For NBS implementation in Germany, Zingraff-Hamed et al. (2021) identified four governance models: Cooperation and incitation, Co-design, Citizen power, and Top-down, which differ according to the diversity of involved stakeholder groups and the direction of mainstreaming at the operational and institutional level. The authors did not identify the “best” governance model as there is no “one-size-fits-all” model. All governance models have in common that they include different stakeholder groups which show that a high degree of cooperation between the stakeholders improves NBS implementation potential. Municipalities, citizens, and NGOs are identified as key groups to be included. Further, local authorities—so-called “local champions”—have a crucial role in integrating NBS into location-based planning.

## Conclusions

The contributions to this Special Issue reflect the emergence of increasing research regarding the effects, planning, and governance of NBS. The Special Issue has shown the broad spectrum of NBS interventions that can help address societal challenges in the case of river landscapes and shed light on approaches for assessing their effects, for integrated planning at local to regional scales, and for initiating governance schemes for successful realization in practice.

The contributions also reflect the need for more research to further advance scientific understanding of how NBS can be harnessed in river landscapes to meet, together with technical solutions, the increasing societal challenges. With respect to the three fields of NBS research outlined above and addressed in the contributions of this special issue, the following avenues for further research emerge:

- How efficient and effective are NBS towards achieving ecological, social, and economic outcomes in different social-ecological context conditions in river landscapes, in particular in comparison to conventional, technical alternatives? How do those effects change over time in response to important drivers of change? And how do NBS effects relate to issues of distributional equity and justice?
- Which approaches for planning and designing NBS can be recommended in different social-ecological settings to help crafting engaging visions of NBS futures, to develop plausible scenario pathways for attaining those visions, and for spatially negotiating the contestations involved in making progress? More knowledge is also needed on how the necessary knowledge integration across sectors and academic and non-academic knowledge holders can best be realized and insights communicated to diverse audiences.
- Which governance models can facilitate NBS implementation in different governance contexts and actor constellations? Which roles can new business models play as part of such governance arrangements? Knowledge is needed on how adequate funding schemes can be put into place, how stewardship for the implementation of NBS can be harnessed, and how the benefits and costs of implementing NBS can be balanced in just ways across affected actor groups.
